# Impact of Nonlocality on Group Delay and Reflective Behavior Near Surface Plasmon Resonances in Otto Structure

**DOI:** 10.3390/nano11071780

**Published:** 2021-07-08

**Authors:** Lin Wang, Shangqing Liang, Yuanguo Zhou, Li-Gang Wang

**Affiliations:** 1 College of Electronics and Information, Hangzhou Dianzi University, Hangzhou 310018, China; 2 College of Communication and Information Engineering, Xi’an University of Science and Technology, Xi’an 710054, China; 3 Zhejiang Province Key Laboratory of Quantum Technology and Device, Department of Physics, Zhejiang University, Hangzhou 310027, China

**Keywords:** nonlocal response, dispersion relation, reflection, group delay, zero and pole

## Abstract

In this work, we study the effects of nonlocality on the optical response near surface plasmon resonance of the Otto structure, and such nonlocality is considered in the hydrodynamic model. Through analyzing the dispersion relations and optical response predicted by the Drude’s and hydrodynamic model in the system, we find that the nonlocal effect is sensitive to the large propagation wavevector, and there exists a critical incident angle and thickness. The critical point moves to the smaller value when the nonlocal effect is taken into account. Before this point, the absorption of the reflected light pulse enhances; however, the situation reverses after this point. In the region between the two different critical points in the frequency scan calculated from local and nonlocal theories, the group delay of the reflected light pulse shows opposite behaviors. These results are explained in terms of the pole and zero phenomenological model in complex frequency plane. Our work may contribute to the fundamental understanding of light–matter interactions at the nanoscale and in the design of optical devices.

## 1. Introduction

Nanophotonics is the study of both the behavior of light at nanoscale and the interaction of submicron objects with light [1,2]. As a multidisciplinary scientific and technical area, it attracts considerable attention in electrical engineering, solid-state physics, physical chemistry, biophysics, and biochemistry. An important branch of nanophotonics is optics and optical engineering based on metal-dielectric nanostructures, whose amazing properties can arise from the collective movement of conduction band electrons tightly bound to a metal–insulator interface called surface plasmons [3]. The constitutive parameters, the dielectric permittivity, magnetic permeability, and electrical conductivity are commonly used to study the optical response of metals [4]. The Drude model [5], which describes the dielectric permittivity of the metal, is linear and local in the space and time domain in electrodynamics, and it has been used in accounting for numerous plasmonic phenomena and experiments. The Drude model performs well for many years, but fails to explain some optical effects in the recent studies, due to ignoring the nonlocality (also called spatial dispersion effect), such as the significant blueshift in plasmon resonances [6,7], the field enhancement of film-coupled nanospheres [8], the size-dependent damping in individual metallic nanoparticles [9], and the multipole plasmon modes at metal surface [10]. As the size of structures is comparable to or smaller than the Fermi wavelength of charge carriers, the impact of nonlocal effect increases. Therefore, the Drude model should be replaced by more advanced descriptions, for instance, the hydrodynamic model.

The hydrodynamic model has been used to account for the spatial dispersion produced from the repulsion between free electrons inside metals [11,12]. This model was first introduced by Bloch in 1933 [13], and it resurfaced again when the implementation based on a derived equation of motion for the hydrodynamic Drude model was presented to study the anomalous absorption of Au nanowires [14]. By contrast with other methods, it clearly suffers from an uncertainty about which additional boundary conditions should be used, but allows for more transparent physical interpretations. Moreover, it can be reasonably implemented in numerical calculations and useful for finding the closed-form, analytical result [15]. Therefore, the hydrodynamic model has attracted considerable attention, and it can be used to study the optical response in the different metallic geometries, such as the field enhancement and extinction in the metallic structures, including plasmonic nanowire dimers [16], silver nanogroove [17] and plasmonic tips [18], the mode confinement of plasmonic waveguides [19], quantum confinement and grain boundary electron scattering in connected gold nanoprisms structures [20], second-harmonic generation enhancement in optical split-ring resonators [21], the size-dependent nonlocal effects in plasmonic semiconductor particles [22], and the dispersion relation in metallo-dielectric multilayer configurations [23,24,25]. Simultaneously, it has been applied to transformation-optics approaches to investigate the optical response of non-trivial plasmonic metasurfaces [26,27]. Up to now, research on the spatial dispersion based on the hydrodynamical mode has concentrated on two directions: (i) developing numerical tools based on the hydrodynamical mode to take account for the phenomena in different structures [15,23,28,29] and (ii) theoretically studying the effect of the spatial dispersion [24,30,31].

In this work, we investigate the influence of the nonlocality on the optical response near the surface plasmon resonance of a typical dielectric medium-coupler configuration. The large wavevector leads to a significant discrepancy between the Drude model (i.e., local theory) and the hydrodynamic model (i.e., nonlocal theory). With reference to previous research available in the literature [23,24,25], we pay attention to the behaviors of the reflected light pulse. By analyzing the movements of zeros and poles of the reflection coefficient in the frequency complex domain, the corresponding optical properties, including the amplitude and group delay of the reflected pulse, are investigated. These results may be beneficial to the design of the optical devices and control the pulse propagation.

The whole paper is organized as follows. In Section 2, we present the theoretical model and calculation of the wave propagation. In Section 3, we discuss the dispersion relation, the amplitude, and group delay of the reflection coefficient in the local and nonlocal theories, respectively. The zero and pole phenomenological model are used to explain the behaviors above. Finally, a conclusion is given in Section 4.

## 2. Materials and Methods

**Figure 1:**
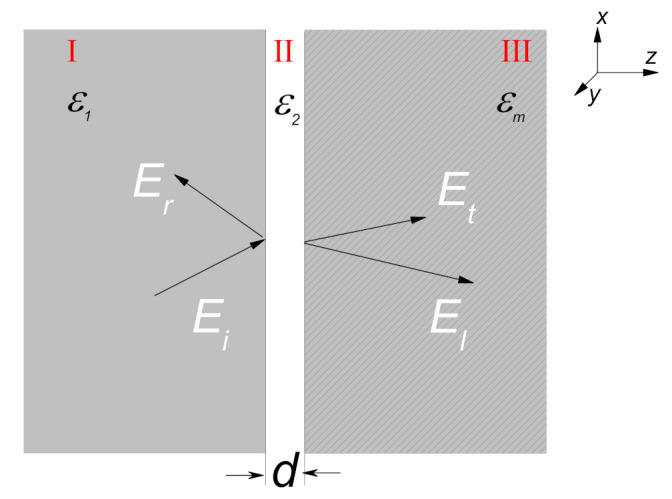
Schematic representation of an Otto structure. The dielectric layer II having small permittivity $\varepsilon_{2}$ and width *d* is sandwiched by the metal $\varepsilon_{m}$ and another dielectric medium I with large permittivity $\varepsilon_{1}$. Here, $E_{i}$ and $E_{r}$ are the incident and reflected light pulses, respectively. $E_{t}$ and $E_{l}$ denote the transmitted transverse and longitudinal wave, respectively.

The structure considered here is represented in Figure 1. Dielectric medium I has large permittivity $\varepsilon_{1}$ and the metal is separated by a gap of width *d*. The relative frequency-independent permittivity of the gap is $\varepsilon_{2}$. Let a transverse-magnetic (TM) plane wave be injected into the layer system from the dielectric medium I at an incident angle θ, and the surface plasmon can be excited under special situations. Here, note that the nonlocality has no impact on *s*-polarization, so we only consider the *p*-polarization [15]. All fields in the system can be described by Maxwell’s equations. Assuming the time dependence is $e^{-i\omega t}$, when the nonlocality is taken into account, the Maxwell’s equations can be expressed as

(1) $$\begin{array}{ccc} ▽\times E & = & i\omega \mu_{0}H, \end{array}$$

(2) $$\begin{array}{ccc} ▿\times H & = & -i\omega \varepsilon_{0}\varepsilon_{m}\left[E-a▿\left(▿\cdot E\right)\right], \end{array}$$

inside the metallic layer. Here, $\varepsilon_{m}=1-\frac{\omega_{p}^{2}}{\omega^{2}+i\gamma \omega }$ is the relative permittivity of the metal, and $a=\frac{\beta^{2}}{\omega_{p}^{2}-\omega^{2}-i\gamma \omega }$, where $\omega_{p}$ is the plasma frequency, γ is the damping factor, and $\beta ≃1.39\times 10^{6}$ m/s is the phenomenological nonlocal parameter proportional to Fermi velocity [15]. From these equations, it can be seen that there are two different waves supported in the metallic layer. In *Case I*, when the divergence of the electric field is zero (▿·E=0), it corresponds to the transverse wave. At this situation, Equation (2) reduces to the usual expression

(3) $$\begin{array}{c} ▿\times H=-i\omega \varepsilon_{0}\varepsilon_{m}E. \end{array}$$

From Equations (1) and (3), the magnetic and electric fields can be written as

(4) $$\begin{array}{ccc} H_{y}^{T} & = & \left(A_{t}e^{ik_{mz}z}+B_{t}e^{-ik_{mz}z}\right)e^{i\left(k_{x}x-\omega t\right)}, \end{array}$$

(5) $$\begin{array}{ccc} E_{x}^{T} & = & \frac{ik_{mz}}{i\omega \varepsilon_{0}\varepsilon_{m}}\left(A_{t}e^{ik_{mz}z}-B_{t}e^{-ik_{mz}z}\right)e^{i\left(k_{x}x-\omega t\right)}, \end{array}$$

(6) $$\begin{array}{ccc} E_{z}^{T} & = & \frac{-ik_{x}}{i\omega \varepsilon_{0}\varepsilon_{m}}\left(A_{t}e^{ik_{mz}z}+B_{t}e^{-ik_{mz}z}\right)e^{i\left(k_{x}x-\omega t\right)}, \end{array}$$

where $A_{t}$ and $B_{t}$ are the amplitudes of transverse mode, $k_{mz}^{2}=k_{0}^{2}\varepsilon_{m}-k_{x}^{2}$, $k_{0}=\omega /c$ is the wavevector in vacuum, and $k_{x}=k_{0}\sin\theta$ is the wavevector component paralleled to the interface. In *Case II*, the curl of the electric field is zero (▿×E=0); it corresponds to the longitudinal wave. The underlying condition behind this case is that the magnetic field in the *y* direction cannot exist, and the relation between the electric fields satisfies $\partial_{x}E_{z}=\partial_{z}E_{x}$. For this case, Equation (2) can also be written as

(7) $$\begin{array}{c} \frac{\partial^{2}}{\partial z^{2}}E_{x}-\left(k_{x}^{2}+\frac{1}{a}\right)E_{x}=0. \end{array}$$

Therefore, the electric fields for the longitudinal wave can be written as

(8) $$\begin{array}{ccc} E_{x}^{L} & = & \left(A_{l}e^{-k_{l}z}+B_{l}e^{k_{l}z}\right)e^{i\left(k_{x}x-\omega t\right)}, \end{array}$$

(9) $$\begin{array}{ccc} E_{z}^{L} & = & \frac{k_{l}}{ik_{x}}\left(-A_{l}e^{-k_{l}z}+B_{l}e^{k_{l}z}\right)e^{i\left(k_{x}x-\omega t\right)}, \end{array}$$

where $A_{l}$ and $B_{l}$ are the amplitudes of longitudinal mode, and the wave vector of the longitudinal electric field is $k_{l}^{2}=k_{x}^{2}+\frac{\omega_{p}^{2}}{\beta^{2}}\left(1+\frac{1}{\chi_{f}}\right)$, where $\chi_{f}=-\frac{\omega_{p}^{2}}{\omega^{2}+i\gamma \omega }$ is the susceptibility of the free electrons. In dielectric medium I, only the transverse wave is supported, and the electric and magnetic fields are

(10) $$\begin{array}{ccc} H_{1y} & = & \left(A_{1}e^{ik_{1z}z}+B_{1}e^{-ik_{1z}z}\right)e^{i\left(k_{x}x-\omega t\right)}, \end{array}$$

(11) $$\begin{array}{ccc} E_{1x} & = & \frac{ik_{1z}}{i\omega \varepsilon_{0}\varepsilon_{1}}\left(A_{1}e^{ik_{1z}z}-B_{1}e^{-ik_{1z}z}\right)e^{i\left(k_{x}x-\omega t\right)}, \end{array}$$

where $k_{1z}^{2}=k_{0}^{2}\varepsilon_{1}-k_{x}^{2}$. The electric and magnetic fields in medium II can be expressed as the similar form

(12) $$\begin{array}{ccc} H_{2y} & = & \left(A_{2}e^{ik_{2z}z}+B_{2}e^{-ik_{2z}z}\right)e^{i\left(k_{x}x-\omega t\right)}, \end{array}$$

(13) $$\begin{array}{ccc} E_{2x} & = & \frac{ik_{2z}}{i\omega \varepsilon_{0}\varepsilon_{2}}\left(A_{2}e^{ik_{2z}z}-B_{2}e^{-ik_{2z}z}\right)e^{i\left(k_{x}x-\omega t\right)}, \end{array}$$

where $k_{2z}^{2}=k_{0}^{2}\varepsilon_{2}-k_{x}^{2}$. According to the boundary condition, the magnetic fields and electric fields are continuous at the interfaces of z=0

(14) $$\begin{array}{ccc} H_{1y}{|}_{z=0} & = & H_{2y}{|}_{z=0}, \end{array}$$

(15) $$\begin{array}{ccc} E_{1x}{|}_{z=0} & = & E_{2x}{|}_{z=0}, \end{array}$$

and z=d

(16) $$\begin{array}{ccc} H_{2y}{|}_{z=d} & = & H_{y}^{T}{|}_{z=d}, \end{array}$$

(17) $$\begin{array}{ccc} E_{2x}{|}_{z=d} & = & E_{x}^{T}{{|}_{z=d}+E_{x}^{L}|}_{z=d}. \end{array}$$

Within the nonlocality theory, an additional boundary condition at the medium II–metal surface is required to determine the amplitude of longitudinal wave. Finally, the reflection coefficient with nonlocality can be derived using the zero entire polarization at the interface $P_{mz}=-\frac{1}{i\omega }\partial_{x}H_{y}^{T}-\varepsilon_{0}\left(E_{z}^{T}+E_{z}^{L}\right)=0$ as the additional boundary condition [15]. It is given by

(18) $$\begin{array}{c} r^{nloc}=\frac{\left(1+\eta^{nloc}\right)\left(1-\alpha \right)+\left(1-\eta^{nloc}\right)\left(1+\alpha \right)e^{ik_{2z}d}}{\left(1+\eta^{nloc}\right)\left(1+\alpha \right)+\left(1-\eta^{nloc}\right)\left(1-\alpha \right)e^{ik_{2z}d}}, \end{array}$$

where $\alpha =k_{2z}\varepsilon_{1}/k_{1z}\varepsilon_{2}$ and $\eta^{nloc}=k_{mz}\varepsilon_{2}/k_{2z}\varepsilon_{m}-i\Omega \varepsilon_{2}/k_{2z}$, here $\Omega =\frac{k_{x}^{2}}{k_{l}}\left(\frac{1}{\varepsilon_{m}}-1\right)$. When Ω=0, ignoring the nonlocality, the reflection coefficient can retrieve to the usual expression

(19) $$\begin{array}{c} r^{loc}=\frac{\left(1+\eta^{loc}\right)\left(1-\alpha \right)+\left(1-\eta^{loc}\right)\left(1+\alpha \right)e^{ik_{2z}d}}{\left(1+\eta^{loc}\right)\left(1+\alpha \right)+\left(1-\eta^{loc}\right)\left(1-\alpha \right)e^{ik_{2z}d}}, \end{array}$$

with $\eta^{loc}=k_{mz}\varepsilon_{2}/k_{2z}\varepsilon_{m}$. Comparing Equation (18) with Equation (19), it can be seen that at normal incidence (i.e., $k_{x}=0$), there is no difference between two reflection coefficients. As $k_{x}$ changes, the effect of nonlocality can be observed. In order to observe the effect of nonlocality experimentally, we can measure the group delay of the light reflected from the Otto structure besides a reflectivity spectrum. Assuming the incident light pulse is a Guassian pulse with a very narrow spectrum, the spectral width of the pulse △ω⩽ω, so that the distortion of the reflected light pulse can be neglected. In this limit, the group delay of the reflected light pulse can be calculated by [32,33,34]

(20) $$\begin{array}{c} \tau_{r}=\frac{d\phi_{r}}{d\omega }, \end{array}$$

where $\phi_{r}$ is the phase of the reflection coefficient, and *r* denotes $r^{loc}$ or $r^{nloc}$. The group delay is the time delay of the pulse envelope as it propagates through a medium [34]. The negative group delay means the advance of the pulse’s peak (or envelope) and corresponds to fast light or superluminal propagation. While the positive group delay means slow light or subluminal propagation.

In the following calculation, we assume $\varepsilon_{1}=9$ for the medium I and that medium II is a vacuum (or air). This means that the critical angle of total reflection is $\theta_{c}\approx 19.48^{\circ }$.

## 3. Numerical Results and Discussion

**Figure 2:**
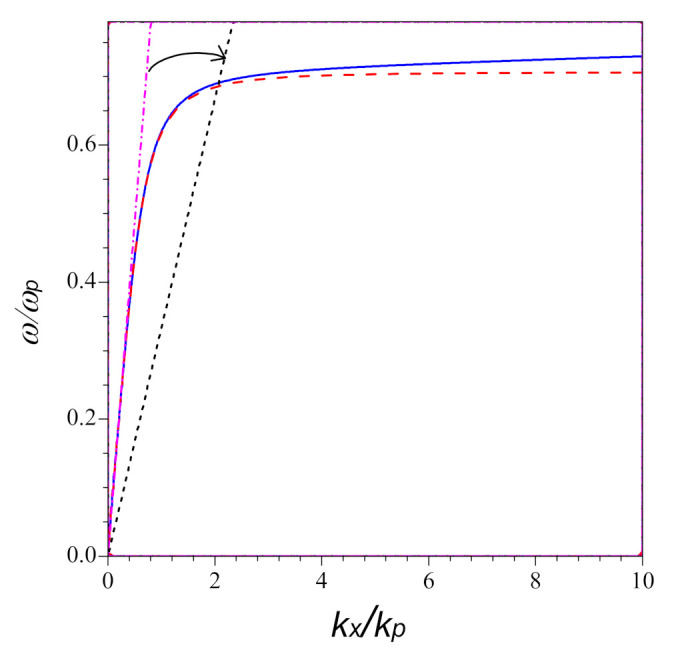
Dispersion curves for the surface plasmon-polarization at the interface between vacuum and metal. The red dashed and blue solid lines correspond to the local and nonlocal cases, respectively. Here, the pink dot-dashed and black dotted lines denote the light cones in the medium I at $\theta =20^{\circ }$ and $\theta =90^{\circ }$, respectively. The arrow points to the direction in which the incident angle increases. The other parameters are $\omega_{p}=1.3926\times 10^{16}$ Hz, $\gamma =3.18712\times 10^{13}$ Hz for Ag, and $\beta =1.39\times 10^{6}$ m/s.

In order to study how nonlocality influences the optical response of the system, the dispersion relations at the interface between medium II and metal within local theory and nonlocal theory are plotted by the red dashed and blue solid lines in Figure 2, respectively. The black dotted and pink dot-dashed lines denote the light cones in the Medium I at different inclined incidence. The arrow indicates the incident angle changing from small to large. From Figure 2, it can be seen that with increasing of $k_{x}$, two dispersion curves increase synchronously for $k_{x}$. However, for large $k_{x}$, they move away from each other. For the local case, the curve tends to a constant, $\frac{\omega_{p}}{\sqrt{2}}$, while as for the nonlocal case, the dispersion curve increases linearly with $k_{x}$. It is clear that the impact of nonlocal effect becomes more sensitive to the large propagation constant $k_{x}$.

The surface plasmon at the interface can be excited once the condition of $k_{x}=k_{0}\sqrt{\frac{\varepsilon_{2}\varepsilon_{m}}{\varepsilon_{2}+\varepsilon_{m}}}$ holds. We can approach this condition by medium I coupled $k_{x}=k_{0}\sqrt{\varepsilon_{1}}\sin\theta$, here the incident angle $\theta >\theta_{c}$. The intersections in Figure 2 predict the resonant coupling between the surface plasmon and the incident light pulse for different angles of incidence. Under the same incident angle, the surface plasmon resonance moves to the higher frequency for the nonlocal case, compared to the case of local effect. If $k_{x}$ is large enough, the difference between the two resonant frequency can increase constantly with increasing of $k_{x}$.

**Figure 3:**
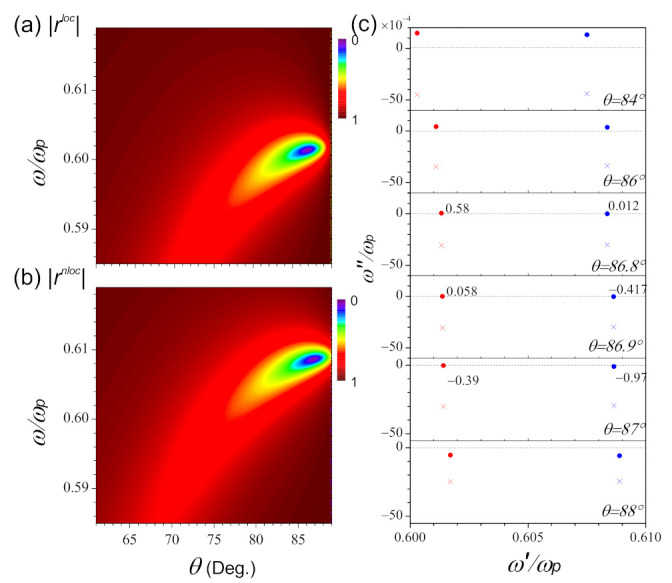
The comparison of the reflection as functions of incident angle θ and frequency in units of $\omega_{p}$ in the frame of (**a**) local and (**b**) nonlocal theories. (**c**) The locations of zero (circle) and pole (cross) in the complex plane of frequency, when the angle changes from $\theta =84^{\circ }$ to $88^{\circ }$. The red and blue color denote the local and nonlocal model, respectively. The thickness of the air gap is d=10 nm, and other parameters are the same in Figure 2.

The amplitudes of the reflected light pulse as functions of the frequency and incident angle in the local and nonlocal model are plotted in Figure 3a,b, respectively. As the width of the air gap reduces to nanoscale d=10 nm, the excitation of the surface plasmon occurs at a larger angle of incidence, approaching 87${}^{\circ }$. Comparing Figure 3b with Figure 3a, the surface plasmon polariton moves to a higher frequency when the nonlocality is taken into account. This is in good agreement with the results in Figure 2. As we mentioned in our previous work, the behaviors of the reflected light pulse can be explained by the frame of zero and pole phenomenological model [35]. Here, we also apply it to the analysis of the nonlocality.

In the frequency complex plane, the zeros ${\tilde{\omega }}_{o}={\omega_{o}}^{\prime }+i{\omega_{o}}^{\prime \prime }$ and poles ${\tilde{\omega }}_{p}={\omega_{p}}^{\prime }+i{\omega_{p}}^{\prime \prime }$ should satisfy the following equations:

(21) $$\begin{array}{c} \left(1+\eta^{loc}\right)\left(1-\alpha \right)+\left(1-\eta^{loc}\right)\left(1+\alpha \right)e^{ik_{2z}d}=0, \end{array}$$

(22) $$\begin{array}{c} \left(1+\eta^{nloc}\right)\left(1-\alpha \right)+\left(1-\eta^{nloc}\right)\left(1+\alpha \right)e^{ik_{2z}d}=0, \end{array}$$

for ${\tilde{\omega }}_{o}^{loc}$ and ${\tilde{\omega }}_{o}^{nloc}$, respectively, and

(23) $$\begin{array}{c} \left(1+\eta^{loc}\right)\left(1+\alpha \right)+\left(1-\eta^{loc}\right)\left(1-\alpha \right)e^{ik_{2z}d}=0, \end{array}$$

(24) $$\begin{array}{c} \left(1+\eta^{nloc}\right)\left(1+\alpha \right)+\left(1-\eta^{nloc}\right)\left(1-\alpha \right)e^{ik_{2z}d}=0, \end{array}$$

for ${\tilde{\omega }}_{p}^{loc}$ and ${\tilde{\omega }}_{p}^{nloc}$, respectively. It is clearly seen that the positions of ${\tilde{\omega }}_{o}$ and ${\tilde{\omega }}_{p}$ can be adjusted by the thickness *d* or the angle of incidence. Figure 3c shows the movements of zeros (circle) and poles (cross) in the frequency complex plane when the incident angle changes from $84^{\circ }$ to $88^{\circ }$. From Figure 3c, it can be seen that for the two cases, as incident angle increases, the zero moves from the upper-half plane, across the real axis (denoted by the dashed line), then to the lower-half plane. The changes of the zero indicate the existence of the critical angle, which corresponds to the case of ${\omega_{o}}^{\prime \prime }=0$, and such characteristics leads to the total absorption of the incident energy by the structure. The critical angle is located between $86.9^{\circ }$∼$87^{\circ }$ for $\left|r^{loc}\right|$, and between 86.8${}^{\circ }$∼$86.9^{\circ }$ for $\left|r^{nloc}\right|$. On the other hand, as θ changes, the poles always shift in the region of ${\omega_{p}}^{\prime \prime }<0$, and never across the real axis. Next, we should investigate the behaviors of the reflected light pulse with the movements of the corresponding singularities.

**Figure 4:**
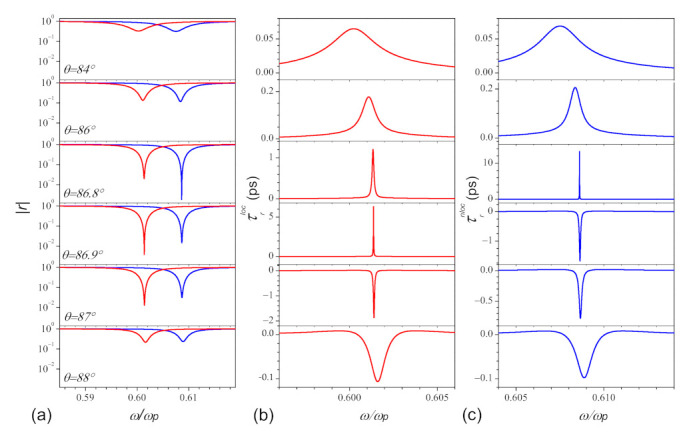
The amplitude (**a**) and group delay (**b**,**c**) of the reflected light pulse as the function of frequency under different incident angle $\theta =84^{\circ }$, $86^{\circ }$, $86.8^{\circ }$, $86.9^{\circ }$, $87^{\circ }$, and $88^{\circ }$ from top to bottom. Here the red and blue color denote the framework of local and nonlocal cases, respectively. Other parameters are the same in Figure 3.

The corresponding optical response of the reflection is plotted in Figure 4. Figure 4a shows the reflection $\left|r^{loc}\right|$ and $\left|r^{nloc}\right|$ under different incident angles $\theta =84^{\circ }$, $86^{\circ }$, $86.8^{\circ }$, $86.9^{\circ }$, $87^{\circ }$, and $88^{\circ }$, from top to bottom. We note that the minimal value and the width of resonance for $\left|r\right|$ are sensitive to the incident angle. As the incident angle increases (or decreases) from the critical angle of incidence $\theta_{opt}$ to the larger (or smaller) angle, the minimal value and the width of resonance increases. From the comparison between local and nonlocal spectra of the reflection, we find that the nonlocality introduces a shift of the critical incident angle, and $\theta_{opt}^{nloc}$ appears ahead of $\theta_{opt}^{loc}$. For the case of $\theta <\theta_{opt}^{nloc}$, the position of $\left|r_{min}^{loc}\right|$ is always higher than $\left|r_{min}^{nloc}\right|$, which means the absorption is weaker than the case with nonlocality; once $\theta >\theta_{opt}^{nloc}$, the situations reverse.

The corresponding group delays of the reflected light pulse are plotted in Figure 4b,c, calculated from the local (red lines) and nonlocal (blue lines) model, respectively. From Figure 4b,c, we find that both the group delays near the resonance are positive, which means the subluminal propagation, if the condition ${\omega_{o}}^{\prime \prime }>0$ holds ($84^{\circ }$∼$86.9^{\circ }$ for $\tau_{r}^{loc}$, and $84^{\circ }$∼$86.8^{\circ }$ for $\tau_{r}^{nloc}$), and it becomes negative corresponding to the superluminal propagation when ${\omega_{o}}^{\prime \prime }<0$ ($87^{\circ }$∼$88^{\circ }$ for $\tau_{r}^{loc}$, and $86.9^{\circ }$∼$88^{\circ }$ for $\tau_{r}^{nloc}$). Therefore, in the region between the two critical incident angles, the group delays of the reflected light pulse, within the local and nonlocal theory, show the opposite behaviors totally (i.e., $\tau_{r}^{loc}>0$ and $\tau_{r}^{nloc}<0$ ).

**Figure 5:**
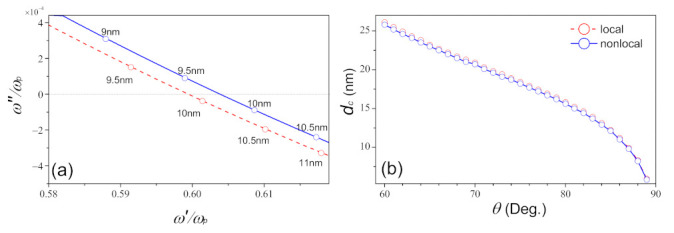
(**a**) The trajectories of zeros in the complex frequency plane as a function of *d* under $\theta =87^{\circ }$. (**b**) The critical thickness $d_{c}$ as a function of incident angle. The red dashed line and blue solid line correspond to the local and nonlocal cases, respectively.

As mentioned above, the movements of zeros in the complex frequency domain can also be controlled by the width of medium II. The trajectories of the zeros in the complex plane as functions of *d* and the critical thickness $d_{c}$ under different incident angles are plotted in Figure 5. As shown in Figure 5a, as *d* increases, ${\tilde{\omega }}_{o}$ shifts from the upper to lower-half plane for both cases. Compared with the local case ($d_{c}\approx 9.9$ nm), the critical thickness $d_{c}$, for which ${\tilde{\omega }}_{o}$ crosses the real axis, appears in advance for the nonlocal case ($d_{c}\approx 9.8$ nm). From Figure 5b, it shows that the critical $d_{c}$ decreases with the increasing of the incident angle, and the result of $d_{c}^{nloc}<d_{c}^{loc}$ is always valid when θ extends into other incident angles.

**Figure 6:**
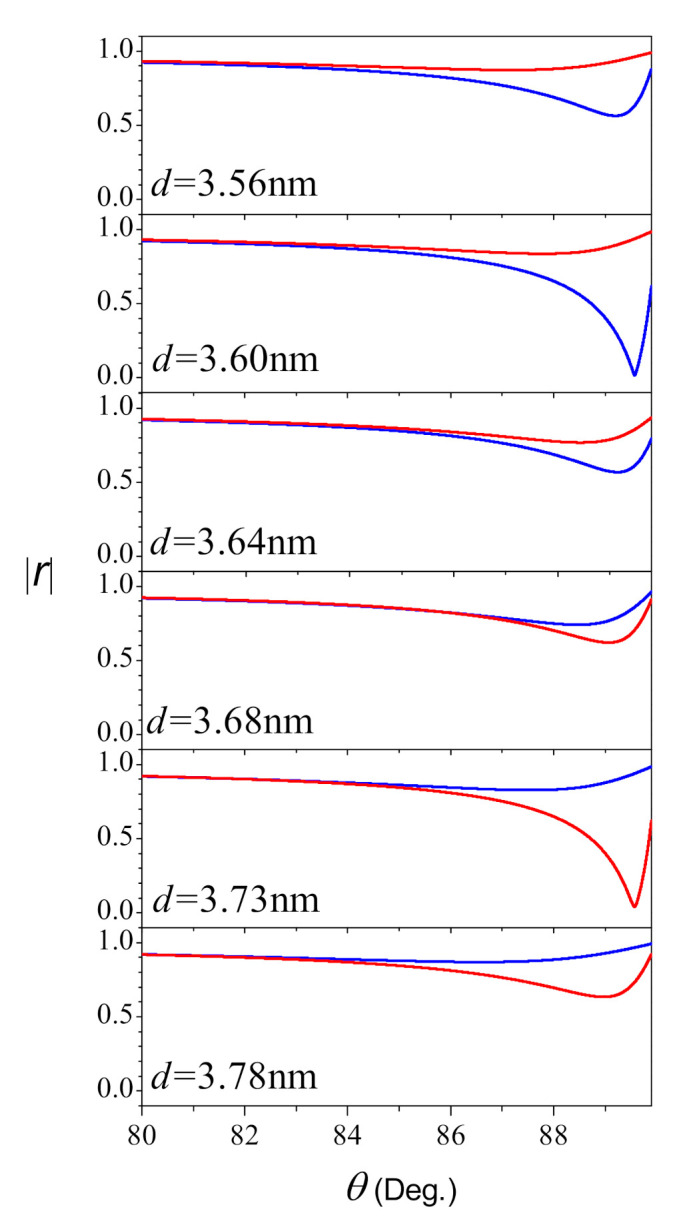
The amplitude of the reflected light pulse as the function of incident angle at λ=488 nm under different thickness. Here, the red and blue color denote the framework of local and nonlocal cases, respectively. Other parameters are the same in Figure 2.

Owing to the enhancement of electromagnetic fields at the metal interface and strong absorption of the reflection, the configuration we considered is useful in biosensing and spectroscopy [36], and the angular measurement approaches are the most widely used. Therefore, the angular scans around the surface plasmon resonance cannot be overlooked in the present work. Here, we choose λ=488 nm as the incident light pulse, and Figure 6 plots the amplitude of the reflected light pulse for different thickness in the range from 3.56 nm to 3.78 nm in the visible range. The red and blue lines calculated in terms of the Drude and hydrodynamical approaches, respectively. From Figure 6 we note that both the angular position and the width of resonance are dependence on the thickness. The decrease in the minimum when *d* varied from 3.56 nm to 3.73 nm for $\left|r_{min}^{loc}\right|$, and 3.56 nm to 3.60 nm for $\left|r_{min}^{nloc}\right|$. The changes completely reversed when the thickness exceeds 3.73 nm for $\left|r_{min}^{loc}\right|$, and 3.60 nm for $\left|r_{min}^{nloc}\right|$. This means the total absorption in the reflection occurred nearly at 3.60 nm when the nonlocal effect taking into account, and almost 3.73 nm without nonlocality. These two special thickness are the critical thickness as well, so there is $d_{c}^{nloc}<d_{c}^{loc}$, which is in total agreement with the results on the frequency scan.

## 4. Conclusions

In summary we have investigated the optical response near the surface plasmon resonance of Otto structure in the frame of Drude’s model and the hydrodynamic model. By contrast, it can be seen that the nonlocal effects is significantly more pronounced to the high wavevector. In the frame of the pole and zero phenomenological model, we found the existence of the critical incident angle (and thickness of the gap layer) for both cases which corresponds to the zero in the real axis. When the zeros of the reflection move from the upper to lower half plane of the complex frequency, the propagation of the reflected light pulse changes from subluminal to superluminal. However, due to the nonlocal effect, the critical points always appears in advance. In addition, in the region between the two different critical points (come from the local and nonlocal cases), the group delays of the reflected light pulse show opposite behaviors. Before the critical incident angle (or thickness) with nonlocality, the absorption of the reflected light pulse was enhanced compared to the case in the local model, it reversed after this point, both on frequency scans and angular scans.

## Data Availability

Data can be available upon request from the authors.
